# Supplementary material for: Pax3 cooperates with Ldb1 to direct local chromosome architecture during myogenic lineage specification
Source: Nat Commun. 2019 May 24;10:2316. doi: 10.1038/s41467-019-10318-6 (PMC6534668; doi:10.1038/s41467-019-10318-6)
Supplement: Supplementary file 1 — Supplementary information [file 41467_2019_10318_MOESM1_ESM.pdf]

Supplementary Information

Pax3 cooperates with Ldb1 to direct local chromosome architecture during myogenic lineage specification

Alessandro Magli, June Baik, Pruthvi Pota, Carolina Ortiz Cordero, Il-Youp Kwak, Daniel J. Garry, Paul E. Love, Brian D. Dynlacht and Rita C.R. Perlingeiro

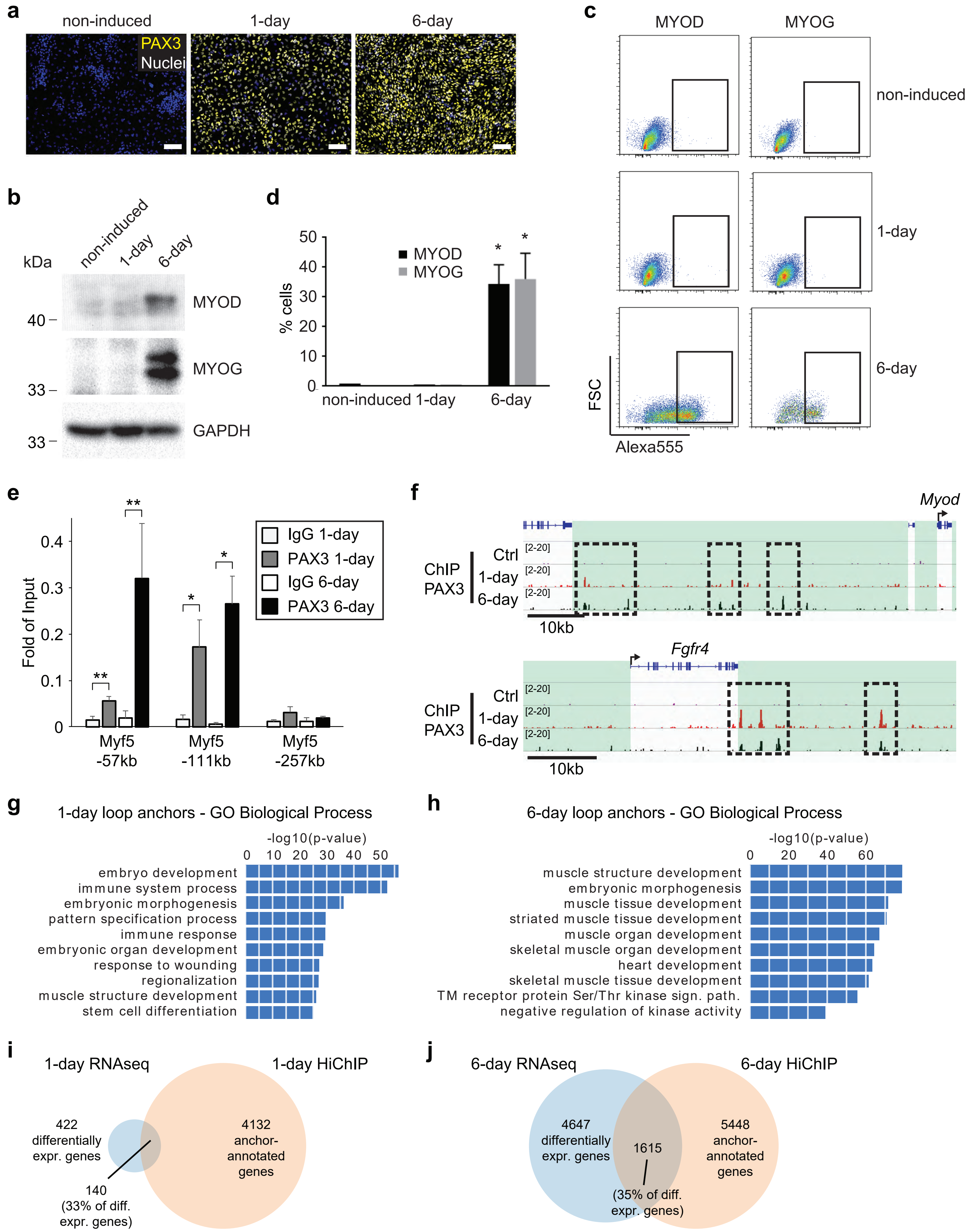

Supplementary Figure 1

Supplementary Figure 1. Dynamics of Pax3-inducible myogenic specification.

(**a**) Immunostaining of Pax3 following 1-day and 6-day induction compared to non-induced cells. PAX3 (yellow); Nuclei (blue). Scale bar: 100µm.

(**b**) Western blot using MYOD and MYOG antibodies in non-induced, 1-day and 6-day iPax3 cells. GAPDH was used as loading control.

(**c-d**) Intracellular staining using MYOD and MYOG antibodies followed by flow cytometry analysis of non-induced, 1-day and 6-day iPax3 cells. FSC: Forward Scatter. MYOD and MYOG (Alexa555). Panel d shows quantification of MYOD+ and MYOG+ cells. Graph represents mean + s.e.m. of n≥3 independent biological replicates. Graph reports frequency of gate 5 from Supplementary Figure 10. Student's *t*-test *p<0.05.

(**e**) qPCR analysis of Pax3 binding at *Myf5* -57kb and -111kb enhancers in 1-day and 6-day Pax3-induced cells. *Myf5* -257kb represents a non-bound control. Graph represents mean + s.e.m. from n≥3 independent experiments. Student's *t*-test *p<0.05, **p<0.01.

(**f**) IGV track displaying Pax3 binding at *Myod* and *Fgfr4* loci in 1-day and 6-day Pax3-induced cells. Dashed black squares indicate Pax3-bound loci. Green boxes represent intergenic regions. Black arrow represents the transcription start site.

(**g-h**) GREAT functional annotation based on Biological Process of loop anchors from 1-day and 6-day Pax3 HiChIP.

(**i-j**) Overlap of genes annotated to 1-day and 6-day Pax3 HiChIP loop anchors with differentially expressed genes identified upon 1-day and 6-day Pax3 induction.

Source data are provided as a Source Data file.

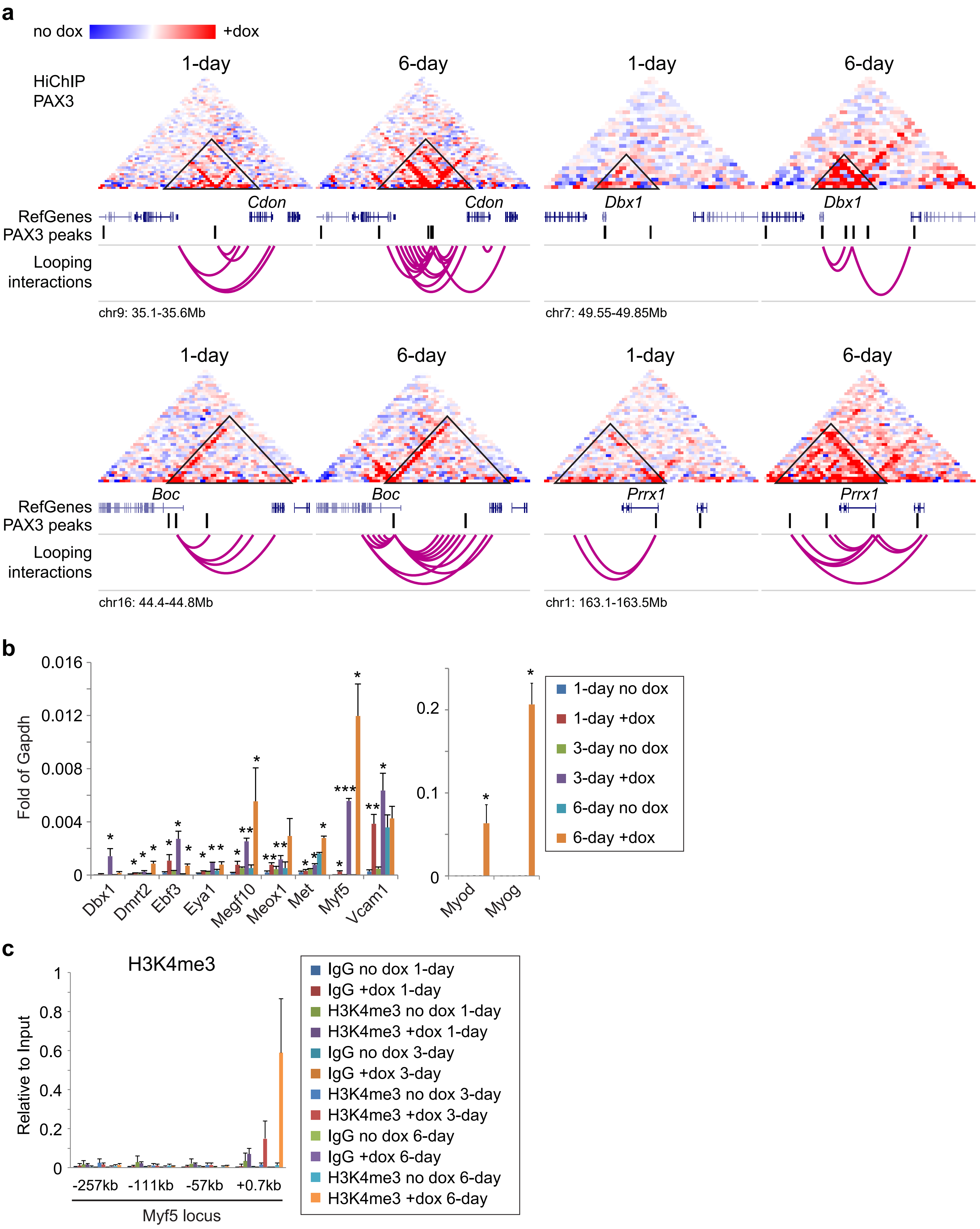

Supplementary Figure 2

Supplementary Figure 2. Time-dependent changes in gene expression, H3K4me3 deposition and looping following Pax3 induction.

(**a**) Long-range interactions at the *Boc*, *Cdon*, *Dbx1* and *Prrx1* loci in 1-day and 6-day Pax3-induced cultures. The contact maps were normalized for sequencing depth and visualized as ratio relative to the background (non-induced Pax3 HiChIP). Triangles indicate domains with long-range chromatin interactions involving Pax3-bound sites (position of Pax3 peaks, genes and chromosome coordinates are showed below). Scale: maxrange = 0.2. Arcs indicate looping interactions identified by FitHiChIP.

(**b**) Gene expression analysis of selected Pax3 target genes in differentiating Pax3-induced (+dox) and non-induced (no dox) ES cells at various time points (1-day, 3-day and 6-day EBs). Graph represents mean + s.d. from (n=3) independent experiments. Student's *t*-test *p<0.05, **p<0.01, ***p<0.001.

(**c**) Analysis of H3K4me3 deposition across the *Myf5* locus in non-induced (no dox) and Pax3-induced (+dox) differentiating ES cells at various time points (1-day, 3-day and 6-day EBs). Graph represents mean + s.d. from (n=3) independent experiments.

Source data are provided as a Source Data file.

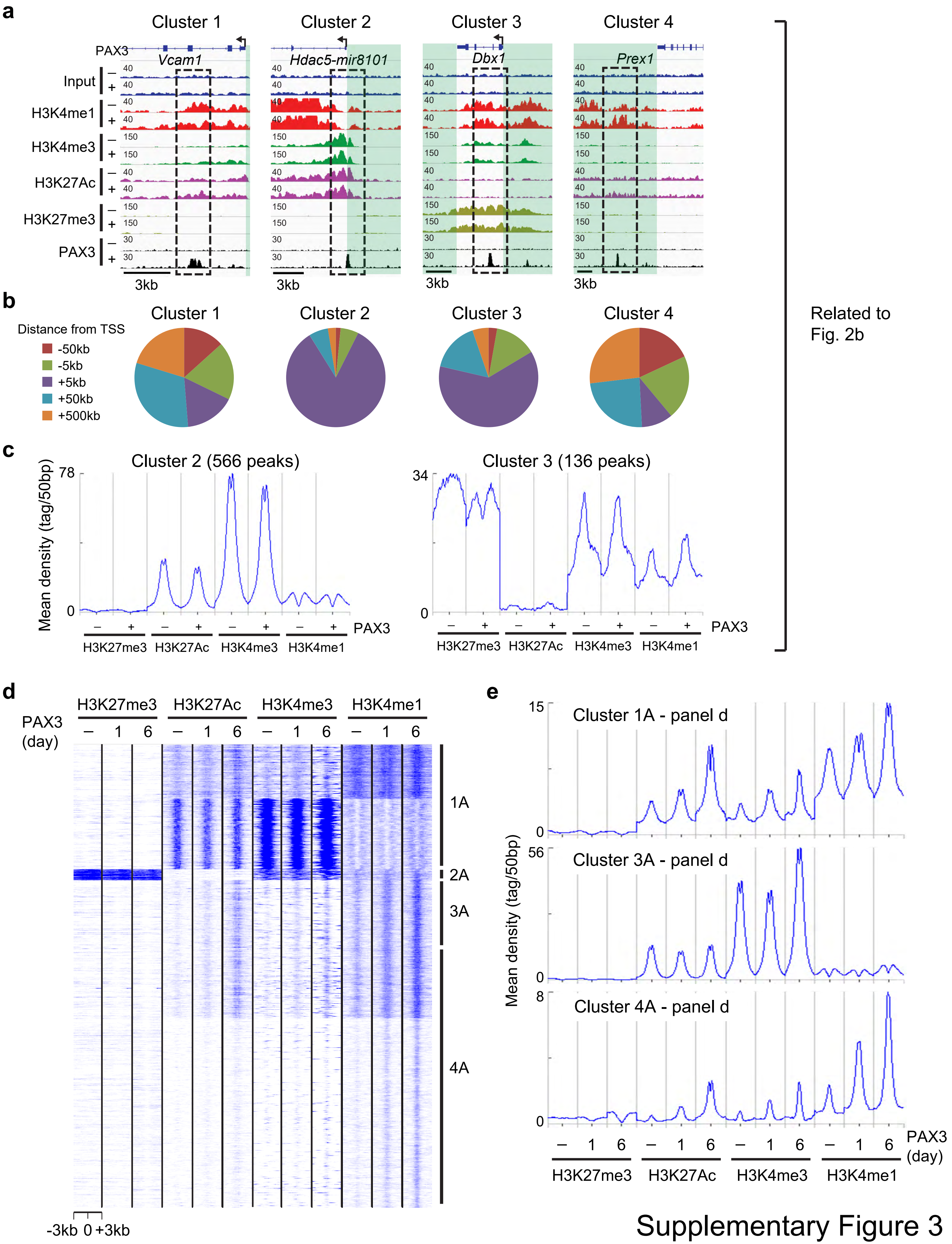

Supplementary Figure 3

Supplementary Figure 3. Pax3 induces chromatin remodeling in mesodermal cells.

(**a**) IGV track displaying H3K4me1, H3K4me3, H3K27Ac, H3K27me3 and Pax3 genomic occupancy at representative Pax3-bound loci from Cluster 1, 2, 3 and 4 in 1-day Pax3-induced (+) and non-induced (-) EBs cultures. Dashed black squares indicate Pax3-bound loci. Green boxes represent intergenic regions. Black arrow represents the transcription start site.

(**b**) Distance (as bins) of Pax3 peaks from the nearest annotated gene.

(**c**) Distribution of H3K4me1, H3K4me3, H3K27me3 and H3K27Ac ChIP-seq reads across the Pax3 peak center ±3kb for Cluster 2 and 4 shown in Figure 2b.

(**d**) k-means clustering of H3K4me1, H3K4me3, H3K27me3 and H3K27Ac ChIP-seq data from 1-day and 6-day Pax3-induced (+) and non-induced (-) EB cultures. Mapped data were used to generate a Density Tag Map centered on 3780 Pax3 peaks ±3kb. Cluster 1: 1010 peaks; Cluster 2: 87 peaks; Cluster 3: 561 peaks; Cluster 4: 2122 peaks.

(**e**) Distribution of H3K4me1, H3K4me3, H3K27me3 and H3K27Ac ChIP-seq reads across the Pax3 peak center ±3kb for Clusters 1A, 3A and 4A shown in panel d.

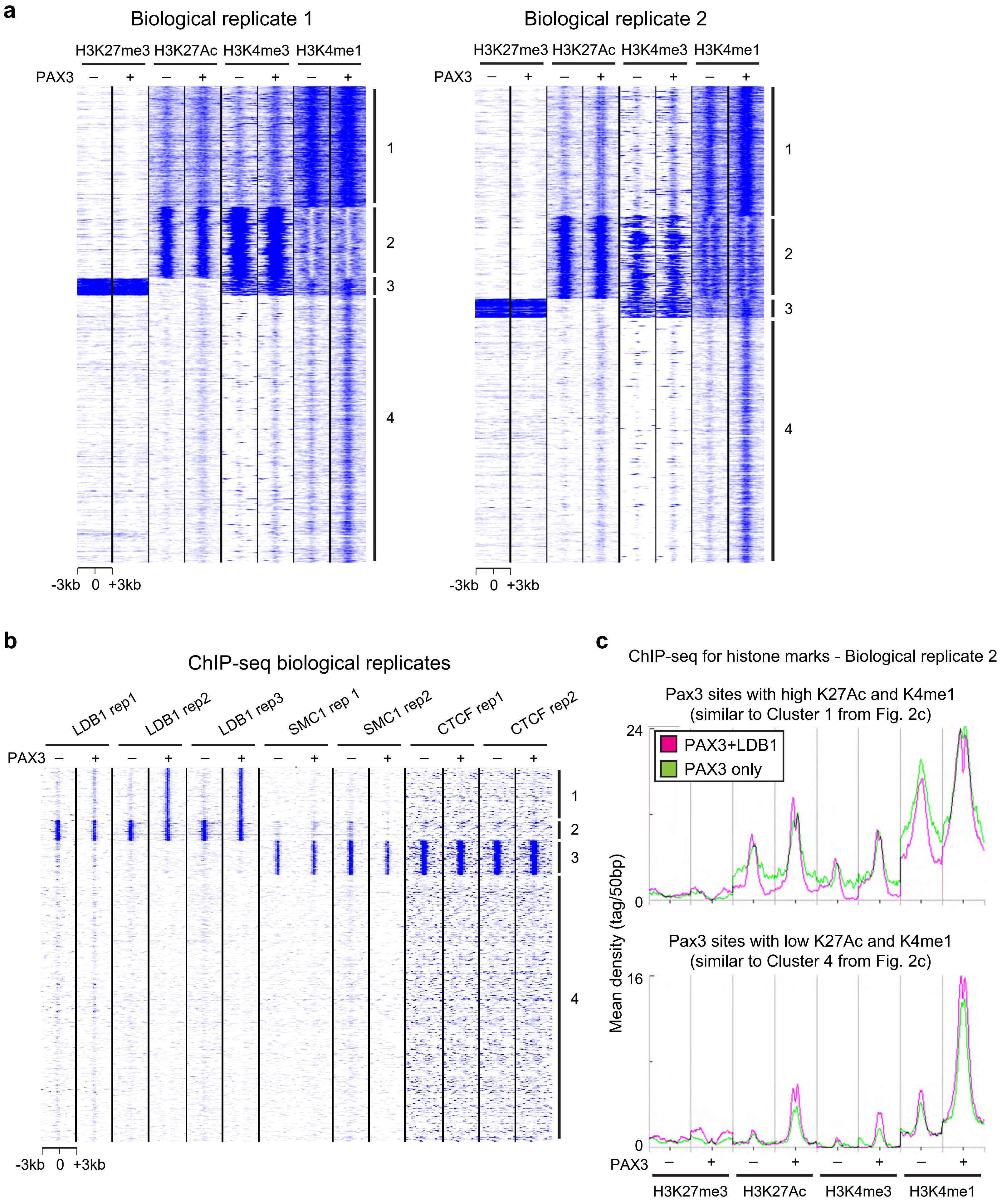

Supplementary Figure 4

Supplementary Figure 4.

(**a**) Replicate 1 and 2 for H3K4me1, H3K4me3, H3K27me3 and H3K27Ac ChIP-seq data from 1-day Pax3-induced (+) and non-induced (-) EB cultures centered on ~3780 PAX3 peaks ±3kb.

(**b**) Replicates for LDB1 (1, 2 and 3), SMC1 (1 and 2) and CTCF (1 and 2) ChIP-seq data from 1-day Pax3-induced (+) and non-induced (-) EB cultures centered on ~3780 PAX3 peaks ±3kb.

(**c**) Replicate 2 showing distribution of ChIP-seq reads for the selected marks at loci characterized by PAX3+LDB1 recruitment (violet) and PAX3 only binding (green).

a

VISTA enhancers overlapping to Pax3 sites

Somite: 9/52
Limb: 25/280
Neural tube: 25/251

b

VISTA somite enhancers overlapping to Pax3 sites

Chrom	Start	End	VISTA_ID
chr1	162074031	162077560	mm712
chr3	99665315	99667144	hs1428
chr4	32301903	32305410	mm720
chr4	108361018	108364528	mm1490
chr5	37825669	37829220	mm426
chr8	25342983	25343992	mm610
chr10	39657714	39664005	hs1690
chr13	60145681	60149199	mm745
chr16	85887133	85888622	hs1684

d

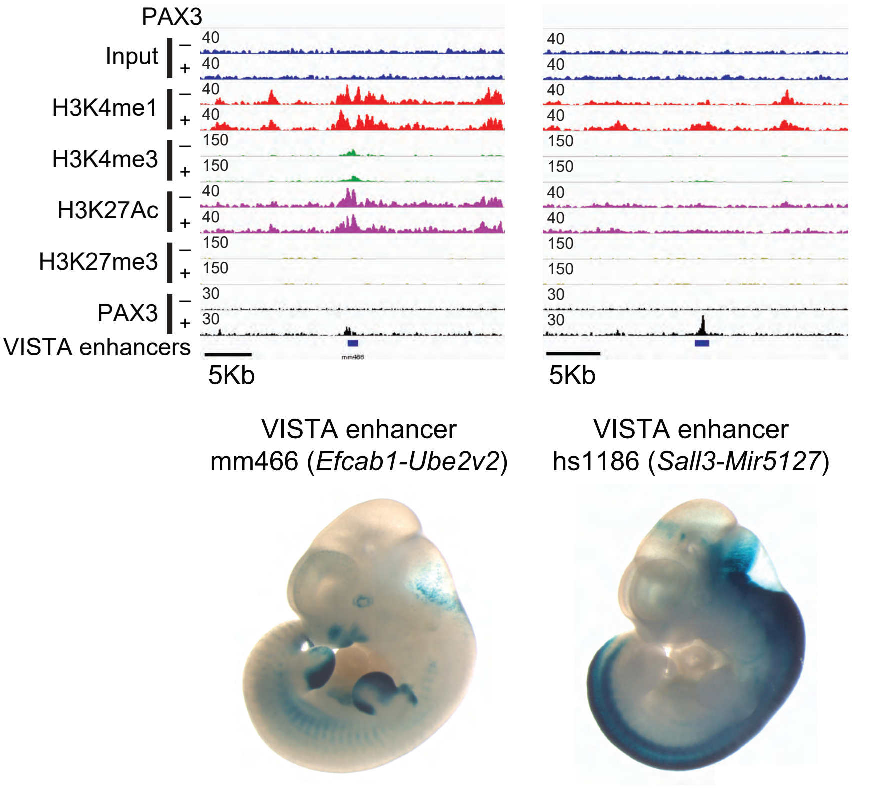

c

VISTA somite enhancers

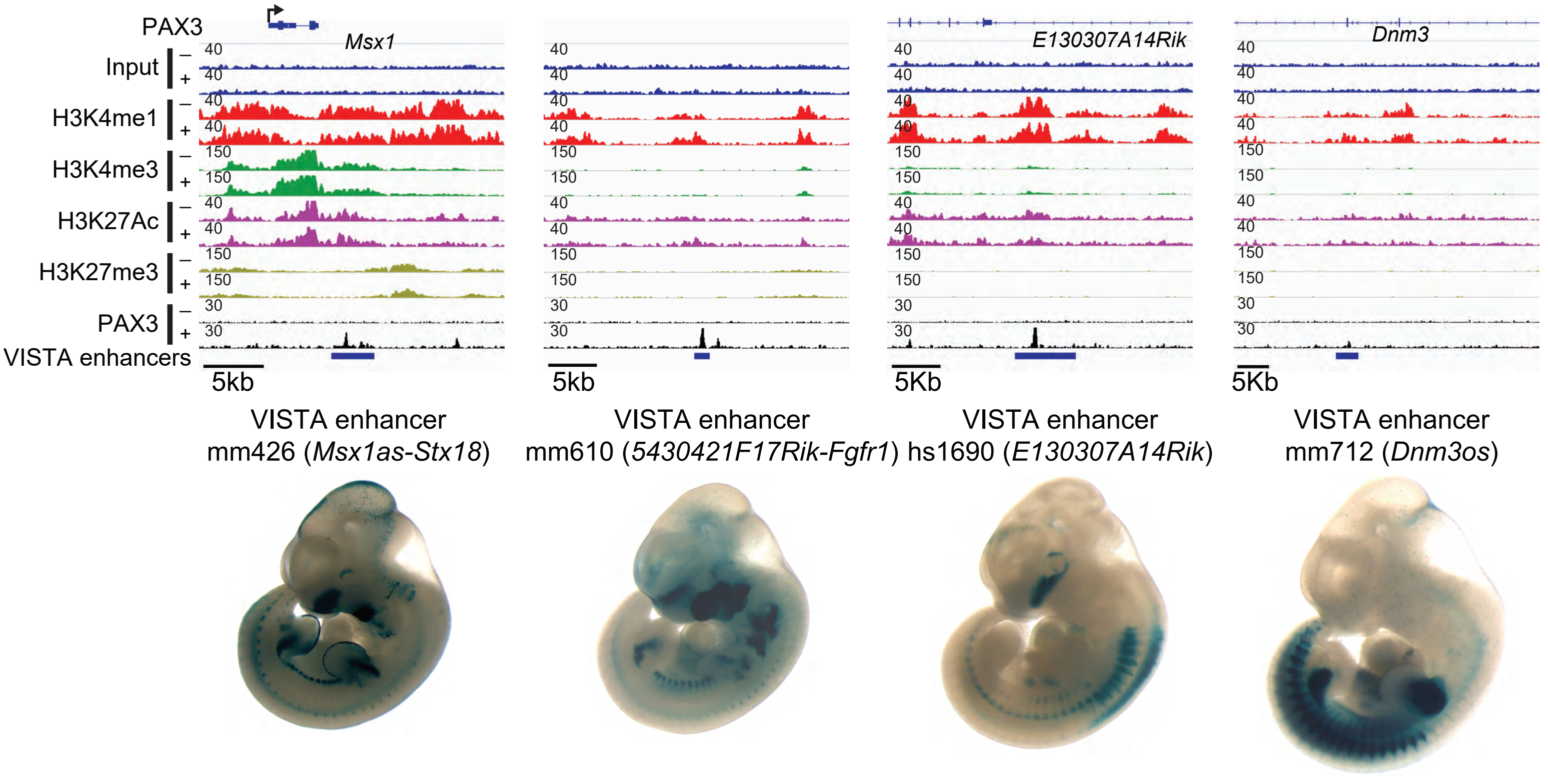

Supplementary Figure 5

Supplementary Figure 5. Pax3 binds elements capable of driving specific gene expression during mouse embryogenesis.

(**a**) Number of Pax3-bound loci overlapping with VISTA enhancer elements annotated to Somite, Limb and Neural tube.

(**b**) List of 9 VISTA enhancers overlapping to 1-day and 6-day Pax3 ChIP-seq peaks.

(**c**) IGV track displaying H3K4me1, H3K4me3, H3K27Ac, H3K27me3 and Pax3 genomic occupancy at selected Pax3-bound loci overlapping with the VISTA enhancer elements mm426 (*Msx1as-Stx18*); mm610 (*5430421F17Rik-Fgfr1*); hs1690 (*E130307A14Rik*) and mm712 (*Dnm3os*). Dashed red squares indicate Pax3-bound loci. Images below represent the E11.5 transgenic embryos expressing LacZ under the control of VISTA elements overlapping to Pax3 peaks. Published images were downloaded from https://enhancer.lbl.gov.

(**d**) Images of IGV tracks (same as for panel c) and E11.5 transgenic embryos expressing LacZ under the control of VISTA elements mm466 (*Efcab1-Ube2v2*) and hs1186 (*Sall3-Mir5127*). These enhancers drive expression respectively in the developing limb and dorsal neural tube. Published images were downloaded from https://enhancer.lbl.gov. Black arrow represents the transcription start site.

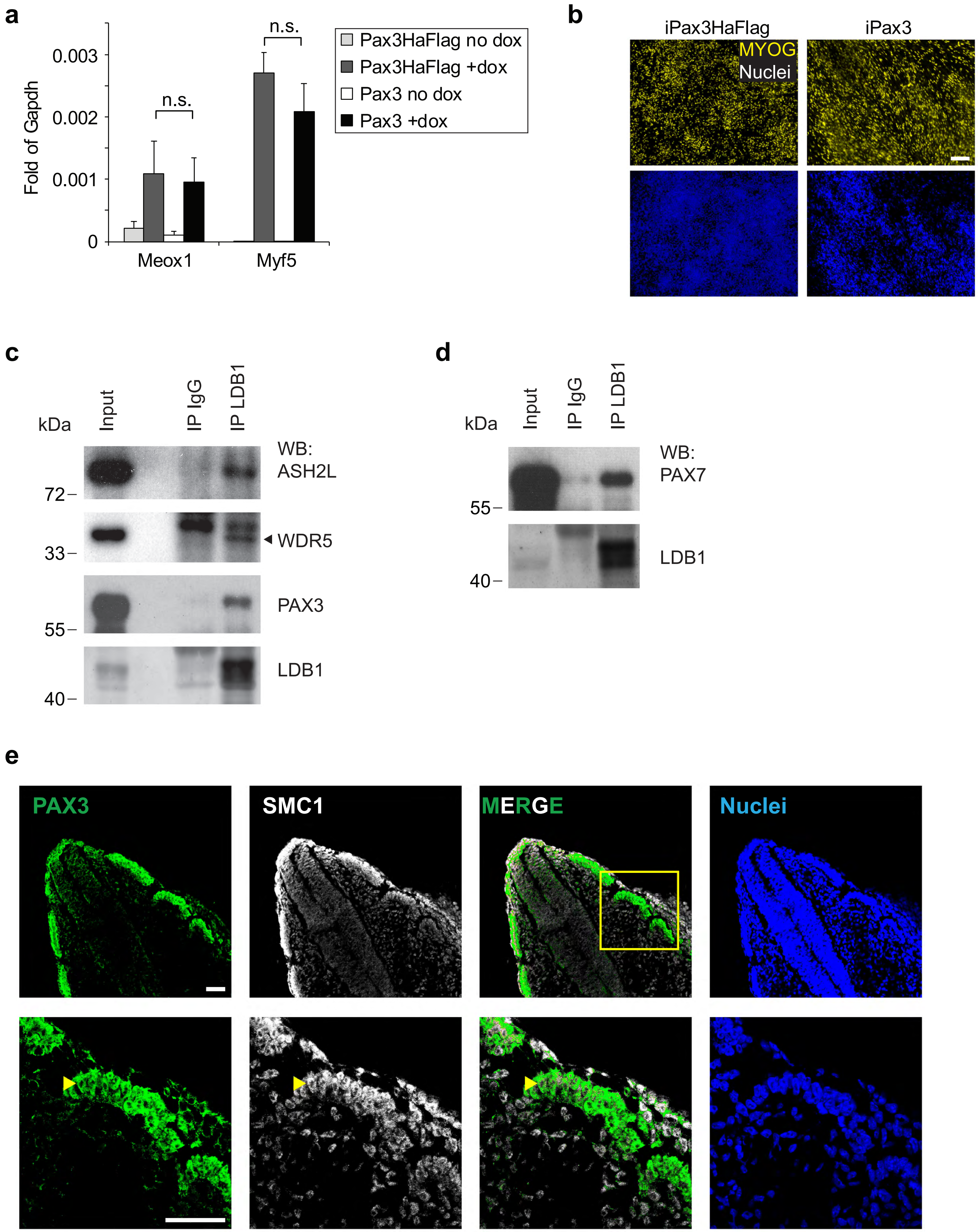

Supplementary Figure 6

Supplementary Figure 6. Co-immunoprecipitation and iPax3HaFlag cell line validation.

(**a**) Gene expression analysis of Meox1 and Myf5 in 1-day induced (+dox) and non-induced (no dox) iPax3HaFlag and iPax3 cells. Graph represents mean + s.d. from independent experiments (n=4). Student's *t*-test n.s. not significant.

(**b**) MYOG immunostaining of 6-day induced cells from iPax3HaFlag and iPax3 cells. MYOG (yellow), Nuclei (blue). Scale bar: 100μm.

(**c**) Co-immunoprecipitation using anti-LDB1 and rabbit IgG control in 1-day induced Pax3 EBs. Western blot was performed using LDB1, ASH2L and WDR5 and PAX3 antibodies. Images are representative of independent biological replicates.

(**d**) Co-immunoprecipitation using anti-LDB1 and rabbit IgG control in 1-day induced Pax7 EBs. Western blot was performed using LDB1 and PAX7 antibodies. Images are representative of independent biological replicates.

(**e**) Immunofluorescence staining shows co-localization of PAX3 and SMC1 in the somites of E9.5 embryos. Upper panel: 20x magnification. Lower panel 63x magnification. PAX3 (green); SMC1 (white); Nuclei (blue). Scale bar: 50μm.

Source data are provided as a Source Data file.

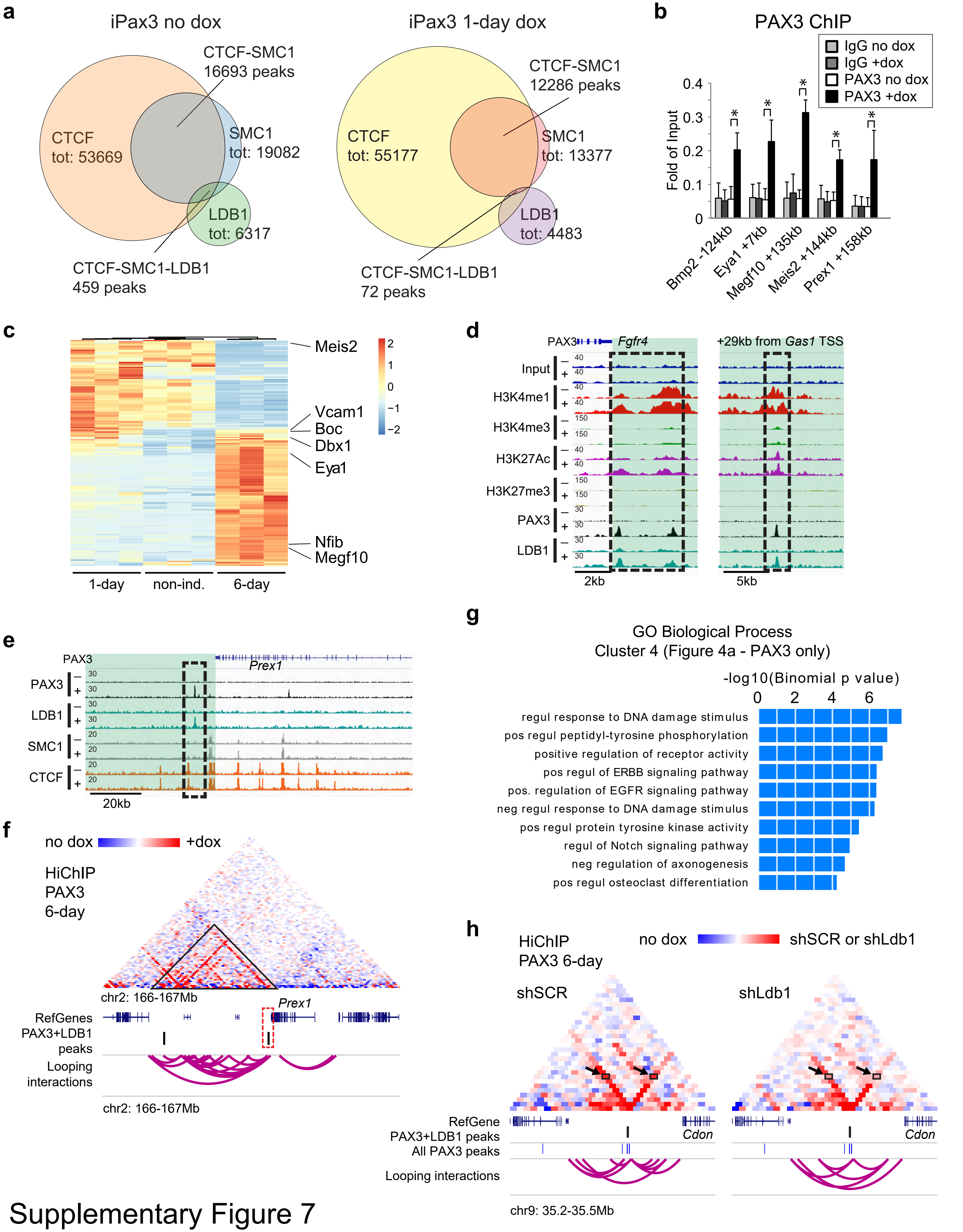

Supplementary Figure 7

Supplementary Figure 7. Ldb1 recruitment at Pax3 sites is associated with enhanced chromatin remodeling and looping.

(**a**) Overlap between CTCF, SMC1 and LDB1 peaks in non-induced and 1-day induced iPax3 cells.

(**b**) qPCR validation of PAX3 binding at selected PAX3 loci in 1-day induced (+dox) and non-induced (no dox) EB cultures. Graph represents mean + s.d. from ($n \geq 3$) independent experiments. Student's *t*-test *$p<0.05$.

(**c**) 185/539 genes annotated to PAX3+LDB1 peaks are significantly up- or down-regulated in Pax3-induced cultures.

(**d**) IGV track displaying H3K4me1, H3K4me3, H3K27Ac, H3K27me3, Pax3 and Ldb1 genomic occupancy at the *Fgfr4* and *Gas1* loci in 1-day Pax3-induced (+) and non-induced (-) EB cultures. Dashed black square indicate Pax3-mediated Ldb1 recruitment. Green boxes represent intergenic regions.

(**e**) IGV track displaying PAX3, LDB1, SMC1 and CTCF genome occupancy at the *Prex1* locus in 1-day Pax3-induced (+) and non-induced (-) EBs. Dashed black square indicate PAX3-mediated LDB1 recruitment. Green boxes represent intergenic regions.

(**f**) HiChIP normalized matrix from 6-day Pax3 induced cells displaying the *Prex1* locus demonstrates long-range interactions involving the 1-day bound PAX3+LDB1 site (dashed black square from panel b). Scale: maxrange = 0.2. Arcs indicate looping interactions identified by FitHiChIP.

(**g**) GREAT functional annotation based on Biological Process of Pax3 only loci (Cluster 4 from Figure 4a).

(**h**) HiChIP normalized matrix from shSCR and shLdb1 6-day Pax3-induced cells displaying the *Cdon* locus. The contact maps were normalized for sequencing depth and visualized as ratio relative to the background (HiChIP in non-induced cells). Black arrows indicate the loss in interaction frequency between 2 loci. Position of PAX3 peaks, genes and chromosome coordinates are showed below the matrix. Scale: maxrange = 0.2. Arcs indicate looping interactions identified by FitHiChIP.

Source data are provided as a Source Data file.

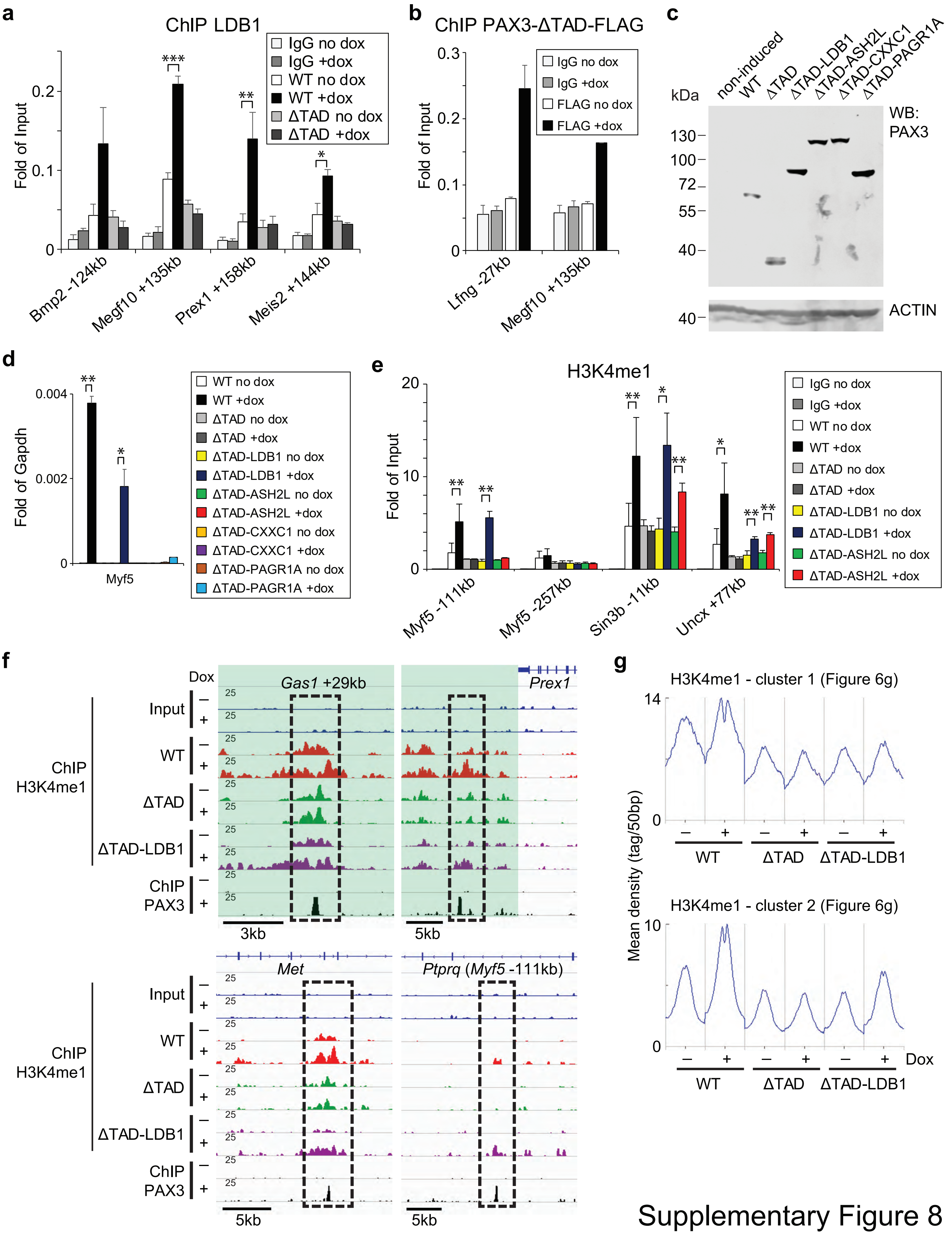

Supplementary Figure 8

Supplementary Figure 8. Forced Ldb1 targeting to Pax3 bound elements induces skeletal myogenesis

(**a**) The PAX3 transactivation domain is required for LDB1 recruitment at PAX3-bound sites. ChIP-qPCR analysis of Ldb1 recruitment at the *Bmp2* -124kb, *Megf10* +135kb, *Prex1* +158kb and *Meis2* +144kb Pax3 bound loci in 1-day WT- and ΔTAD-induced (+) and non-induced (-) EBs cultures. Graph represents mean + s.e.m. from (n=4) independent experiments. Student's *t*-test *p<0.05, **p<0.01, ***p<0.001.

(**b**) ChIP-qPCR analysis using FLAG antibody of ΔTAD-FLAG binding in 1-day induced (+) and non-induced (-) EBs cultures. Graph represents mean + s.d. from (n=2) independent experiments.

(**c**) Western blot analysis confirming dox-dependent expression of WT, ΔTAD, ΔTAD-LDB1, ΔTAD-ASH2L, ΔTAD-CXXC1 and ΔTAD-PAGR1A proteins using an antibody specific for the PAX3 N-terminal region. ACTIN was used as loading control.

(**d**) Gene expression analysis of Myf5 following 1-day induction of WT, ΔTAD, ΔTAD-LDB1, ΔTAD-ASH2L, ΔTAD-CXXC1 and ΔTAD-PAGR1A proteins. Graph represents mean + s.e.m. of n≥3 independent biological replicates. Student's *t*-test *p<0.05, **p<0.01, ***p<0.001.

(**e**) ChIP-qPCR for H3K4me1 deposition at selected loci in 1-day induced (+) and non-induced (-) WT, ΔTAD, ΔTAD-LDB1 and ΔTAD-ASH2L lines. Graph represents mean + s.e.m. from n≥4 independent experiments. Student's *t*-test *p<0.05, **p<0.01.

(**f**) IGV track displaying ChIP-seq data for H3K4me1 in 1-day induced (+) and non-induced (-) WT, ΔTAD and ΔTAD-LDB1 lines. Pax3 genomic occupancy is shown below. Induction of Pax3 WT and ΔTAD-LDB1 induces increase in H3K4me1 deposition at the *Gas1* +29kb, *Met* +98kb and *Myf5* -111kb elements. Dashed black squares indicate PAX3-bound loci. Green boxes represent intergenic regions.

(**g**) Distribution of H3K4me1 ChIP-seq reads across the PAX3 peak center ±3kb for Clusters 1 and 2 shown in Figure 6g.

Source data are provided as a Source Data file.

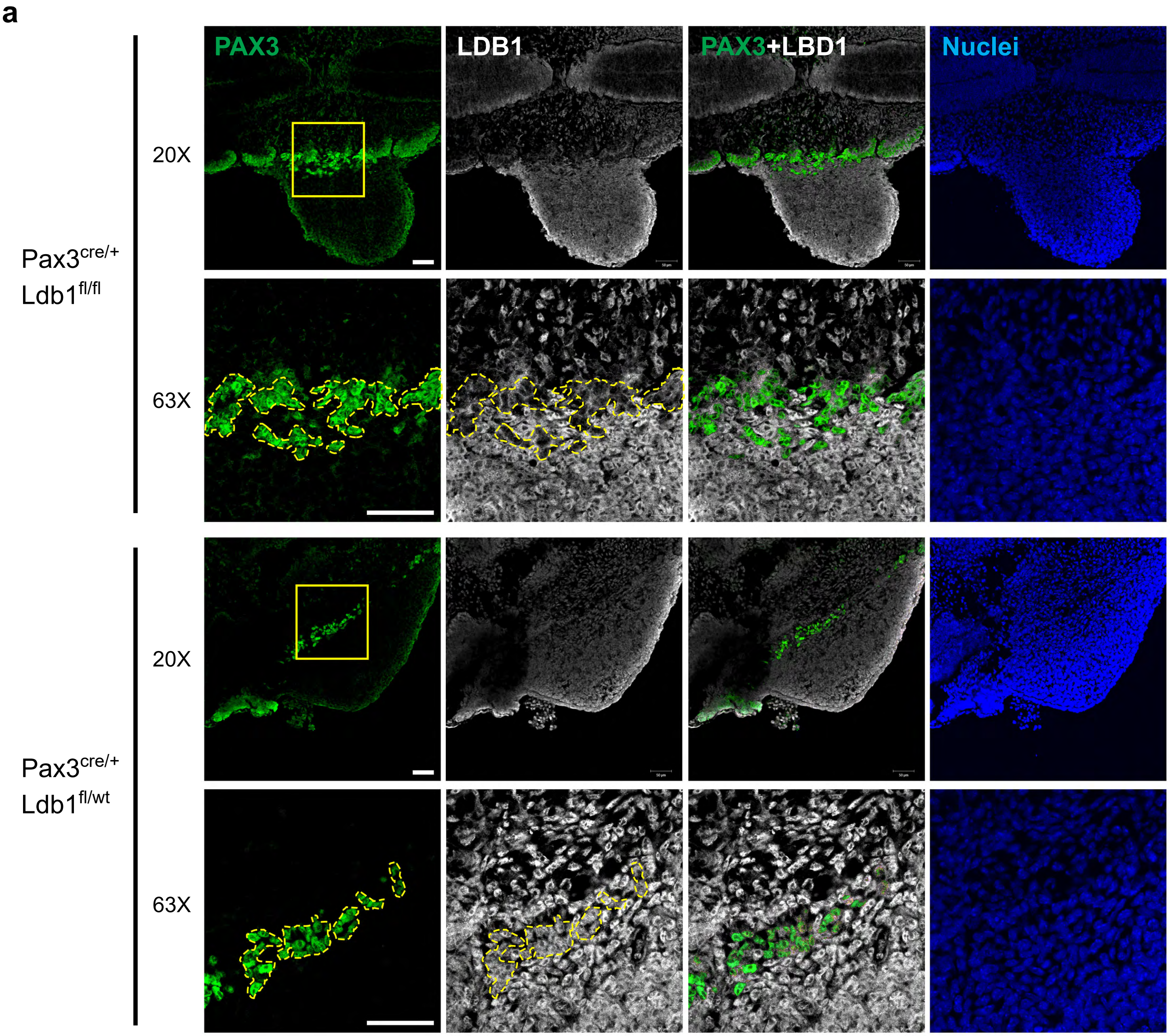

Supplementary Figure 9

Supplementary Figure 9. Ldb1 is required for hypaxial myogenesis.

(**a**) Immunostaining of cryosections from E9.5 Ldb1-deleted embryos shows successful Ldb1 deletion in PAX3+ cells (dashed yellow line) from $Pax3^{cre/+};Ldb1^{fl/fl}$. PAX3 (green); LDB1 (white); nuclei (blue). Magnification 20x and 63x. Bar: 100µm.

a

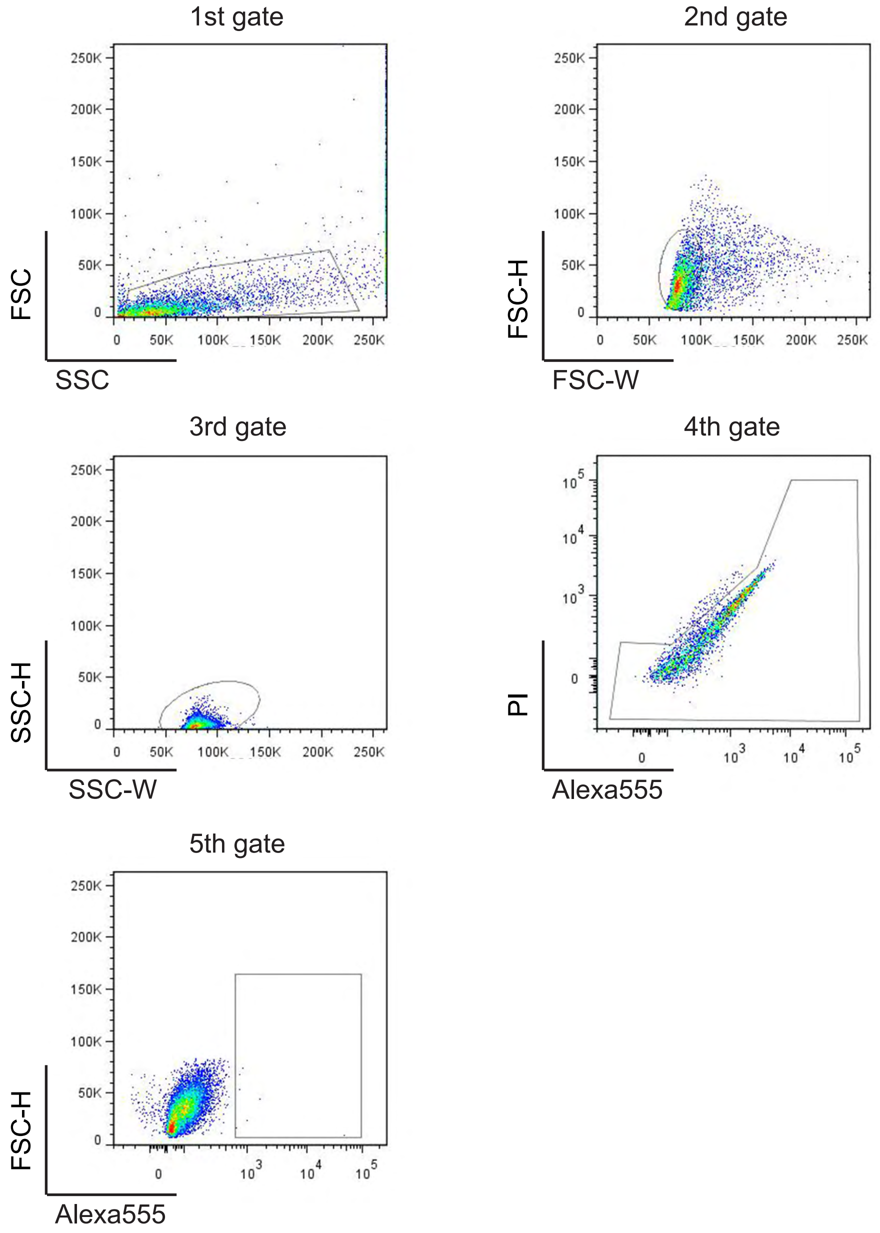

Supplementary Figure 10

Supplementary Figure 10.

(**a**) Gating strategy from flow cytometry analysis of MYOD and MYOG expression reported in Supplementary Figure 1d. Gates are numbered based on the hierarchy followed to determine frequency of the Alexa555+ cells.

Supplementary Table 1. Primer sequences.

Primers for cloning

G-block HaFlag	gatacgagtcgctttcgtgatatttctaaatactttctcgcaacctgaga gcggccgcagaattcGAGAATTTGTATTTTCAGGGTaactacaacatccctac caccggcggcggcggcggctacccatacgatgttccagattacgctctaggca aggtcGACTACAAAGACGATGACGACAAGTAGgctagcgcggccgc cgctgggaccttaagccgacatgtccgtagtacgatgaactaagttttag

Primers for qPCR

Bmp2-124Kb-FW	GAGGCCTTGTCTGTCCCTTC
Bmp2-124Kb-RV	CAGCATTGCTCTGGAGGTCA
Dbx1+2Kb-FW	ACCAACTCTGTGGGAAACGG
Dbx1+2Kb-RV	ACATTTGAGGCGGTTCCCTT
Eya1+7Kb-FW	ACCCTATGATGTCGGCTCAC
Eya1+7Kb-RV	GCTGCCGGCCTTATCCATT
Fgfr4+16Kb-FW	CACACGCATCCATGTTTGGC
Fgfr4+16Kb-RV	CGTGCTGTGTTAACGGTCAG
Fgfr4+19Kb-FW	AAGCTTTGCTGCTCCGTCAA
Fgfr4+19Kb-RV	AACCGGGAGAGACTAAGGGG
Gas1+29Kb-FW	TTGAAGCTTTAACGGCGTGC
Gas1+29Kb-RV	CTTGCTGACACGGGGCATAA
Megf10+135Kb-FW	GTGGGAATGCACTGGCTTTG
Megf10+135Kb-RV	GAAGCAGGCTGGTCACAGAT
Meis2+144Kb-FW	TCACCCTGACAGAAGCAAGG
Meis2+144Kb-RV	TGCAGCCCTTTTCATGCCTA
Met+98Kb-FW	AGTGTTGTGGTTTCACACCG
Met+98Kb-RV	TTCCTGTTACCAGCTCGCC
Myf5+0.7Kb-FW	ACACGGCTCTTAAAGCAATGG
Myf5+0.7Kb-RV	AACTGCTCTGACGGCATGGTA
Myf5-57Kb-FW	ATACAGACATGCAGGCTTCAC
Myf5-57Kb-RV	CTCCGTATGTTTGTTGGAAAGG
Myf5-111Kb-FW	GCCCCTGGTTTTCACAATAA
Myf5-111Kb-RV	GATGGATGGGAAAGATGACC
Myf5-257Kb-FW	GTGTGTCAGTGCATAGCCTAA
Myf5-257Kb-RV	AGGAAGAGCTTGATGGACCAA
Nkx2.5-19Kb-FW	CGGTTCCATCCTTGACCACTC
Nkx2.5-19Kb-RV	GGATCTGGGATCTTTCACCCC
Prex1+158Kb-FW	TGTGGATCTGAGAATCCGCTG
Prex1+158Kb-RV	TTGCAGATTTCACGCACACG
Sin3b-10Kb-FW	ACAAGCCTGAACCACAGAGG
Sin3b-10Kb-RV	GCCCACTGTGAGTGGTAAGT
Smarca2+156Kb-FW	GGAGCCACATGACCATGACA
Smarca2+156Kb-RV	CTCAGAAAGCCCAGGCATGA

Supplementary Table 2. List of Antibodies used in this study.

Primary Ab	Application	Species	Cat no	Supplier	Dilution	Notes
ACTIN	WB	mouse monoclonal	MAB1501	EMD Millipore	1:2000	
ASH2L	WB	rabbit polyclonal	A300-489A	Bethyl	1:2000	
CTCF	ChIP	rabbit polyclonal	07-729	EMD Millipore	1:100	
GAPDH	WB	mouse monoclonal	ab8245	Abcam	1:2000	
H3 (pan)	ChIP	rabbit polyclonal	ab1791	Abcam	1:100	
H3K4me1	ChIP	rabbit polyclonal	ab8895	Abcam	1:100	
H3K4me3	ChIP	rabbit polyclonal	ab8580	Abcam	1:100	
H3K27Ac	ChIP	rabbit polyclonal	ab4729	Abcam	1:100	
H3K27me3	ChIP	rabbit polyclonal	07-449	EMD Millipore	1:100	
LDB1	Immunostaining - ChIP - CoIP	rabbit polyclonal	ab96799	Abcam	IF 1:200 - ChIP 1:100 - CoIP 1:100	
LDB1	WB - ChIP	goat polyclonal	sc-11198	Santa Cruz Biotechnology	WB 1:2000 - ChIP 1:100	this antibody has been discontinued
LDB1	ChIP	mouse monoclonal	sc-365074	Santa Cruz Biotechnology	1:100	
MYF5	Immunostaining	rabbit polyclonal	sc-302	Santa Cruz Biotechnology	1:200	this antibody has been discontinued
MYHC embryonic	WB	mouse monoclonal	F1-652	Developmental Studies Hybridoma Bank	1:100 (non-concentrated stock)	
MYHC (pan)	Immunostaining	mouse monoclonal	MF20	Developmental Studies Hybridoma Bank	1:50 (non-concentrated stock)	
MYOD	WB - FACS	mouse monoclonal	554130	BD Biosciences	WB 1:1000 - FACS 1:100	
MYOG	WB - Immunostaining - FACS	mouse monoclonal	F5D	Developmental Studies Hybridoma Bank	WB 1:100 - IF 1:50 - FACS 1:100 (non-concentrated stock)	
PAX3 (C-term directed)	WB - Immunostaining	mouse monoclonal	Pax3	Developmental Studies Hybridoma Bank	WB 1:2000 - IF 1:100	
PAX3	ChIP	goat polyclonal	sc-34926	Santa Cruz Biotechnology	1:100	this antibody has been discontinued
PAX3 (N-term directed)	WB	mouse monoclonal	MAB2457	R&D Systems	1:1000	for analysis of Pax3 ΔTAD fusion proteins
PAX7	WB	mouse monoclonal	Pax7	Developmental Studies Hybridoma Bank	1:1000	
SMC1	Immunostaining - WB - ChIP	rabbit polyclonal	A300-055A	Bethyl	1:2000	
WDR5	WB	rabbit polyclonal	A302-429A	Bethyl	1:2000	
**Controls and secondary Ab**						
Normal mouse IgG	ChIP		sc-2025	Santa Cruz Biotechnology	1:100	
Normal rabbit IgG	ChIP		sc-2027	Santa Cruz Biotechnology	1:100	
Normal goat IgG	ChIP		ab37373	Abcam	1:100	
Alexa-555 anti-mouse	Immunostaining - FACS		A-21424	Invitrogen	IF 1:250 - FACS 1:500	
HRP-conjugated anti-mouse	WB		NA931V	GE Healthcare	1:10000	
HRP-conjugated anti-rabbit	WB		NA934VS	GE Healthcare	1:10000	
HRP-conjugated anti-goat	WB		sc-2020	Santa Cruz Biotechnology	1:10000	
