# Supplementary material for: Pax3 cooperates with Ldb1 to direct local chromosome architecture during myogenic lineage specification
Source: Nat Commun. 2019 May 24;10:2316. doi: 10.1038/s41467-019-10318-6 (PMC6534668; doi:10.1038/s41467-019-10318-6)
Supplement: Supplementary file 2 — Description of Additional Supplementary Files [file 41467_2019_10318_MOESM2_ESM.pdf]

Legends for Supplementary Data 1-5.

Supplementary Data 1. Genomic coordinates (mm10) of looping interactions detected using FitHiChIP in all HiChIP datasets.

Supplementary Data 2. List of genes annotated to loop anchors and comparison with list of 1-day and 6-day differentially expressed genes.

Supplementary Data 3. Annotation of ChIP-seq peaks from Fig. 2b and Supplementary Figure 7a.

Supplementary Data 4. List of proteins identified upon mass spectrometry analysis of the Pax3 interactome.

Supplementary Data 5. Analysis of protein-protein interaction using STRING.
