# Supplementary material for: Pax3 cooperates with Ldb1 to direct local chromosome architecture during myogenic lineage specification
Source: Nat Commun. 2019 May 24;10:2316. doi: 10.1038/s41467-019-10318-6 (PMC6534668; doi:10.1038/s41467-019-10318-6)
Supplement: Supplementary file 9 — Reporting Summary [file 41467_2019_10318_MOESM9_ESM.pdf]

nature research

Corresponding author(s): Rita CR Perlingeiro
Alessandro Magli

Last updated by author(s): Apr 16, 2019

Reporting Summary

Nature Research wishes to improve the reproducibility of the work that we publish. This form provides structure for consistency and transparency in reporting. For further information on Nature Research policies, see Authors & Referees and the Editorial Policy Checklist.

Statistics

For all statistical analyses, confirm that the following items are present in the figure legend, table legend, main text, or Methods section.

n/a	Confirmed	
☐	☒	The exact sample size (*n*) for each experimental group/condition, given as a discrete number and unit of measurement
☐	☒	A statement on whether measurements were taken from distinct samples or whether the same sample was measured repeatedly
☐	☒	The statistical test(s) used AND whether they are one- or two-sided<br>*Only common tests should be described solely by name; describe more complex techniques in the Methods section.*
☒	☐	A description of all covariates tested
☒	☐	A description of any assumptions or corrections, such as tests of normality and adjustment for multiple comparisons
☐	☒	A full description of the statistical parameters including central tendency (e.g. means) or other basic estimates (e.g. regression coefficient) AND variation (e.g. standard deviation) or associated estimates of uncertainty (e.g. confidence intervals)
☐	☒	For null hypothesis testing, the test statistic (e.g. *F*, *t*, *r*) with confidence intervals, effect sizes, degrees of freedom and *P* value noted<br>*Give P values as exact values whenever suitable.*
☒	☐	For Bayesian analysis, information on the choice of priors and Markov chain Monte Carlo settings
☒	☐	For hierarchical and complex designs, identification of the appropriate level for tests and full reporting of outcomes
☒	☐	Estimates of effect sizes (e.g. Cohen's *d*, Pearson's *r*), indicating how they were calculated

Our web collection on statistics for biologists contains articles on many of the points above.

Software and code

Policy information about availability of computer code

Data collection	FACS Diva (BD bioscience); Zen (Zeiss); Sequest (Thermo Fisher Scientific);
Data analysis	Genomic data analysis: bowtie/2.3.4.1; bedtools/2.27.1; samtools/1.9 ;macs/1.4.1; qeseq/0.2.2; kent/2.4.7; PAVIS2; seqminer/1.3.3; GREAT (Bejerano lab); HiC-pro/2.7.7; FitHiChIP; HiTC; R/3.5.2; Rstudio/1.1.456; pheatmap/1.0.12<br>Protein analysis: STRING (Swiss-prot)<br>Image analysis: Fiji (ImageJ)<br>Statistical analysis: Excel 2016

For manuscripts utilizing custom algorithms or software that are central to the research but not yet described in published literature, software must be made available to editors/reviewers. We strongly encourage code deposition in a community repository (e.g. GitHub). See the Nature Research guidelines for submitting code & software for further information.

Data

Policy information about availability of data

All manuscripts must include a data availability statement. This statement should provide the following information, where applicable:

- Accession codes, unique identifiers, or web links for publicly available datasets
- A list of figures that have associated raw data
- A description of any restrictions on data availability

Sequencing data from this work are publicly available under the GEO accession numbers GSE126362 and GSE125203. The mass spectrometry proteomics data have been deposited to the ProteomeXchange Consortium via the PRIDE partner repository with the dataset identifier PXD012693. Figures 3, 5, 6, 7 and Supplementary figures 1, 2, 6, 7, 8 have associated raw data (already uploaded as source data). Supplementary tables 1, 2, 3 and 4 contains additional list of loci, differentially expressed genes, genomic loci and peptides used for the conclusions of this study. The series PXD012693 will be publicly available in the next few days and can be momentarily accessed using the following key: Username: reviewer94505@ebi.ac.uk Password: JbqbnSfu.

Field-specific reporting

Please select the one below that is the best fit for your research. If you are not sure, read the appropriate sections before making your selection.

☒ Life sciences ☐ Behavioural & social sciences ☐ Ecological, evolutionary & environmental sciences

For a reference copy of the document with all sections, see nature.com/documents/nr-reporting-summary-flat.pdf

Life sciences study design

All studies must disclose on these points even when the disclosure is negative.

Sample size	No sample size calculation was performed. Sample size was determined based on our own experience with the techniques used in this study. Based on the standard deviation of our experimental observations, we are confident the sample size chosen was sufficient to elicit differences between control and treated group.
Data exclusions	No data were excluded
Replication	ChIP-seq and HiChIP experiments were replicated by using an independent differentiation experiment. In some cases, samples were collected on different days and processed at the same time. ChIP-seq for H3K4me1 using dTAD lines was performed once but selected loci were validated in independent biological replicates. Similarly, gene expression analyses were performed on independent samples. Number of replicates for each experiment is reported in the relative figure legend.
Randomization	No randomization involved in this study. We used doxycycline-inducible ES cell line and samples are divided in treated (e.g. +dox) and non-treated (e.g. no dox). Experiments with embryos involved analysis of littermates with different genotypes. We ensured that comparisons would involve embryos characterized by a similar developmental stage defined by counting the number of somite pairs for each embryo.
Blinding	Investigators were not blinded during sample collection and analysis.

Reporting for specific materials, systems and methods

We require information from authors about some types of materials, experimental systems and methods used in many studies. Here, indicate whether each material, system or method listed is relevant to your study. If you are not sure if a list item applies to your research, read the appropriate section before selecting a response.

Materials & experimental systems

n/a	Involved in the study	
☐	☒	Antibodies
☐	☒	Eukaryotic cell lines
☒	☐	Palaeontology
☐	☒	Animals and other organisms
☒	☐	Human research participants
☒	☐	Clinical data

Methods

n/a	Involved in the study	
☐	☒	ChIP-seq
☐	☒	Flow cytometry
☒	☐	MRI-based neuroimaging

Antibodies

Antibodies used

ACTIN MAB1501 EMD Millipore
ASH2L A300-489A Bethyl
CTCF 07-729 EMD Millipore
GAPDH ab8245 Abcam
H3 (pan) ab1791 Abcam
H3K4me1 ab8895 Abcam
H3K4me3 ab8580 Abcam
H3K27Ac ab4729 Abcam
H3K27me3 07-449 EMD Millipore
LDB1 ab96799 Abcam
LDB1 sc-11198 Santa Cruz Biotechnology
LDB1 sc-365074 Santa Cruz Biotechnology
MYF5 sc-302 Santa Cruz Biotechnology
MYHC embryonic F1-652 Developmental Studies Hybridoma Bank
MYHC (pan) MF20 Developmental Studies Hybridoma Bank
MYOD 554130 BD Biosciences
MYOG F5D Developmental Studies Hybridoma Bank
PAX3 (C-term directed) Pax3 Developmental Studies Hybridoma Bank
PAX3 sc-34926 Santa Cruz Biotechnology

	PAX3 (N-term directed) MAB2457 R&D Systems<br>PAX7 Pax7 Developmental Studies Hybridoma Bank<br>SMC1 A300-055A Bethyl<br>WDR5 A302-429A Bethyl
Validation	ACTIN, validated by WB<br>ASH2L, validated by WB<br>CTCF, validated by ChIP-qPCR<br>GAPDH, validated by WB<br>H3 (pan), validated by WB<br>H3K4me1, validated by ChIP-qPCR<br>H3K4me3, validated by ChIP-qPCR<br>H3K27Ac, validated by ChIP-qPCR<br>H3K27me3, validated by ChIP-qPCR<br>LDB1 Abcam, validated by WB<br>LDB1 Santa Cruz, validated by WB<br>LDB1 Santa Cruz, validated by WB<br>MYF5, validated by IF on embryo sections<br>MYHC embryonic, validated by WB<br>MYHC (pan), validated by WB<br>MYOD, validated by WB<br>MYOG, validated by WB<br>PAX3 (C-term directed), validated by WB and IF on embryo sections<br>PAX3 Santa Cruz, validated by WB and ChIP-qPCR<br>PAX3 (N-term directed), validated by WB<br>PAX7, validated by ChIP-qPCR and WB<br>SMC1, validated by WB<br>WDR5, validated by WB

Eukaryotic cell lines

Policy information about cell lines

Cell line source(s)	A2lox-cre, engineered from E14-tg2a mouse embryonic stem cells
Authentication	not authenticated
Mycoplasma contamination	cells are mycoplasma negative
Commonly misidentified lines<br>(See ICLAC register)	none

Animals and other organisms

Policy information about studies involving animals; ARRIVE guidelines recommended for reporting animal research

Laboratory animals	Mus musculus, Pax3-cre, males, age 2-4 months old<br>Mus musculus, Ldb1-flox, females, age 2-4 months old<br>Mus musculus, Pax3-cre,:Ldb1-flox, males, age 2-4 months old
Wild animals	not involved in this study
Field-collected samples	not involved in this study
Ethics oversight	All animals were handled in strict accordance with good animal practice as defined by the relevant national and/or local animal welfare bodies, and all animal work was approved by the University of Minnesota Institutional Animal Care and Use Committee (protocol number 1702-34580A).

Note that full information on the approval of the study protocol must also be provided in the manuscript.

ChIP-seq

Data deposition

☒ Confirm that both raw and final processed data have been deposited in a public database such as GEO.

☒ Confirm that you have deposited or provided access to graph files (e.g. BED files) for the called peaks.

Data access links<br>*May remain private before publication.*	GSE126362: Go to https://www.ncbi.nlm.nih.gov/geo/query/acc.cgi?acc=GSE126362 and enter token udydmcgylfurhap into the box;<br>GSE125203: publicly available
Files in database submission	GSE126362 series:

Files in database submission

d9_plus_H327me3_S6_R1_001.fastq.gz
d9_plus_H3K27Ac_S5_R1_001.fastq.gz
d9_plus_H3K4me1_S3_R1_001.fastq.gz
d9_plus_H3K4me3_S4_R1_001.fastq.gz
dTAD-Ldb1_no_H3K4me1_S9_R1_001.fastq.gz
dTAD-Ldb1_plus_H3K4me1_S10_R1_001.fastq.gz
dTAD_no_H3K4me1_S7_R1_001.fastq.gz
dTAD_plus_H3K4me1_S8_R1_001.fastq.gz
H3K27Ac_no_4_S4_R1_001.fastq.gz
H3K27Ac_no_A4_S20_R1_001.fastq.gz
H3K27Ac_plus_9_S9_R1_001.fastq.gz
H3K27Ac_plus_A9_S24_R1_001.fastq.gz
H3K27me3_no_5_S5_R1_001.fastq.gz
H3K27me3_plus_10_S10_R1_001.fastq.gz
H3K4me1_no_2_S2_R1_001.fastq.gz
H3K4me1_no_A2_S18_R1_001.fastq.gz
H3K4me1_plus_7_S7_R1_001.fastq.gz
H3K4me1_plus_A7_S22_R1_001.fastq.gz
H3K4me3_no_3_S3_R1_001.fastq.gz
H3K4me3_no_A3_S19_R1_001.fastq.gz
H3K4me3_plus_8_S8_R1_001.fastq.gz
H3K4me3_plus_A8_S23_R1_001.fastq.gz
d4_H3K27me3_no_S7_R1_001.fastq.gz
d4_H3K27me3_plus_S8_R1_001.fastq.gz
Input_no_17_CGTACG_L001_R1_001.fastq.gz
Input_no_17_CGTACG_L002_R1_001.fastq.gz
Input_no_17_S85_R1_001.fastq.gz
Input_no_1_S1_R1_001.fastq.gz
Input_no_A1_S17_R1_001.fastq.gz
Input_no_S85_R1_001.fastq.gz
Input_plus_21_ATGAGC_L001_R1_001.fastq.gz
Input_plus_21_ATGAGC_L002_R1_001.fastq.gz
Input_plus_21_S78_R1_001.fastq.gz
Input_plus_6_S6_R1_001.fastq.gz
Input_plus_A6_S21_R1_001.fastq.gz
Input_plus_S88_R1_001.fastq.gz
LDB1_A4_no_S86_R1_001.fastq.gz
LDB1_A4_plus_S89_R1_001.fastq.gz
LDB1_C9_no_S87_R1_001.fastq.gz
LDB1_C9_plus_S90_R1_001.fastq.gz
LDB1_no_28_S41_R1_001.fastq.gz
LDB1_plus_32_S42_R1_001.fastq.gz
CTCF_no_12_S12_R1_001.fastq.gz
CTCF_plus_15_S15_R1_001.fastq.gz
Ctcf_no_19_GGTAGC_L001_R1_001.fastq.gz
Ctcf_no_19_GGTAGC_L002_R1_001.fastq.gz
Ctcf_no_19_S90_R1_001.fastq.gz
Ctcf_plus_23_CACGAT_L001_R1_001.fastq.gz
Ctcf_plus_23_CACGAT_L002_R1_001.fastq.gz
Ctcf_plus_23_S80_R1_001.fastq.gz
SMC1_no_12_S39_R1_001.fastq.gz
SMC1_plus_15_S40_R1_001.fastq.gz
Smc1_no_18_GAGTGG_L001_R1_001.fastq.gz
Smc1_no_18_GAGTGG_L002_R1_001.fastq.gz
Smc1_no_18_S88_R1_001.fastq.gz
Smc1_plus_22_CAACTA_L001_R1_001.fastq.gz
Smc1_plus_22_CAACTA_L002_R1_001.fastq.gz
Smc1_plus_22_S79_R1_001.fastq.gz
Smc1_no_26_CATTTT_L001_R1_001.fastq.gz
Smc1_no_26_CATTTT_L002_R1_001.fastq.gz
Smc1_no_26_S83_R1_001.fastq.gz
Smc1_plus_30_CTCAGA_L001_R1_001.fastq.gz
Smc1_plus_30_CTCAGA_L002_R1_001.fastq.gz
Smc1_plus_30_S87_R1_001.fastq.gz
HiChIP_d4_plus_S1_R1_001.fastq.gz
HiChIP_d4_plus_S1_R2_001.fastq.gz
HiChIP_myog_prog_A_S2_R1_001.fastq.gz
HiChIP_myog_prog_A_S2_R2_001.fastq.gz
HiChIP_myog_prog_B_S3_R1_001.fastq.gz
HiChIP_myog_prog_B_S3_R2_001.fastq.gz
01_d4_Pax3_plusdox_S15_R1_001.fastq.gz
01_d4_Pax3_plusdox_S15_R2_001.fastq.gz
02_d9_Pax3_shSCR_A_S16_R1_001.fastq.gz
02_d9_Pax3_shSCR_A_S16_R2_001.fastq.gz
03_d9_Pax3_shSCR_B_S17_R1_001.fastq.gz

03_d9_Pax3_shSCR_B_S17_R2_001.fastq.gz
04_d9_Pax3_shLdb1_A_S18_R1_001.fastq.gz
04_d9_Pax3_shLdb1_A_S18_R2_001.fastq.gz
05_d9_Pax3_shLdb1_B_S19_R1_001.fastq.gz
05_d9_Pax3_shLdb1_B_S19_R2_001.fastq.gz
06_d4_Pax3_nodox_S20_R1_001.fastq.gz
06_d4_Pax3_nodox_S20_R2_001.fastq.gz
dTAD_Ldb1_nodox_S1_R1_001.fastq.gz
dTAD_Ldb1_nodox_S1_R2_001.fastq.gz
dTAD_Ldb1_plusdox_A_S2_R1_001.fastq.gz
dTAD_Ldb1_plusdox_A_S2_R2_001.fastq.gz
dTAD_Ldb1_plusdox_B_S3_R1_001.fastq.gz
dTAD_Ldb1_plusdox_B_S3_R2_001.fastq.gz

Genome browser session
(e.g. UCSC)

no session generated

Methodology

Replicates

3 replicates for SMC1, LDB1 and PAX3 ChIP-seq in non-induced and 1-day PAX3-induced cells. 2 replicates for CTCF, H3K4me1, H3K4me3, H3K27me3, H3K27Ac ChIP-seq in non-induced and 1-day PAX3-induced cells. 2 replicates for PAX3 ChIP-seq in 6-day PAX3-induced cells. 1 replicate for H3K4me1 ChIP in non-induced and 1-day dTAD proteins-induced cells. 1 replicate for H3K4me1, H3K4me3, H3K27me3 and H3K27Ac ChIP-seq in 6-day PAX3-induced cells. 2 replicates for all HiChIP experiments, excepted for non-induced samples (only replicate for PAX3 no dox and dTAD-LDB1 no dox).

Sequencing depth

sample_name sequenced_reads mapped_reads_noMt %mapped
Input_no_17 48423062 34979340 72.2
Input_plus_21 43363192 35616620 82.1
Pax3_no_20 42628343 35091856 82.3
Input_no_25 36645358 27614494 75.4
Pax3_plus_24 33462267 29904664 89.4
Input_plus_29 49934520 32328966 64.7
Pax3_no_27 31556290 25875446 82.0
Pax3_plus_31 32978701 27057425 82.0
Input_no_11 41172666 35219444 85.5
Input_plus_14 44821767 38397981 85.7
PAX3_no_13 42425547 34822399 82.1
PAX3_plus_16 41700654 35025934 84.0
Input_P3_A 45128522 37289637 82.6
Input_P3_B 44841433 37194520 82.9
Pax3_P3_A 42278158 34419563 81.4
Pax3_P3_B 40976813 33793923 82.5
Smc1_no_18 51633698 43310638 83.9
Smc1_plus_22 43976613 36981183 84.1
Smc1_no_26 34237999 28079378 82.0
Smc1_plus_30 36779295 30506465 82.9
SMC1_no_12 67469154 55526109 82.3
SMC1_plus_15 72041395 59486482 82.6
Ctcf_no_19 33493821 28079378 83.8
Ctcf_plus_23 30065812 25364517 84.4
CTCF_no_12 39032046 32889441 84.3
CTCF_plus_15 42563159 35391531 83.2
LDB1_no_28 86216373 68491305 79.4
LDB1_plus_32 78049540 61943392 79.4
LDB1_A4_no 37430468 36425639 97.3
LDB1_A4_plus 43303473 42161087 97.4
LDB1_C9_no 43232080 42030020 97.2
LDB1_C9_plus 39953994 38855215 97.2
Input_no_1 52746938 44264788 83.9
H3K4me1_no_2 44045499 40235187 91.3
H3K4me3_no_3 45838399 35619656 77.7
H3K27Ac_no_4 45236254 41160711 91.0
H3K27me3_no_5 52023990 40301557 77.5
Input_plus_6 52810203 44485295 84.2
H3K4me1_plus_7 48800479 44988308 92.2
H3K4me3_plus_8 47096097 36545413 77.6
H3K27Ac_plus_9 51522984 47419545 92.0
H3K27me3_plus_10 51450600 38853197 75.5
H3K4me1_no_A2 34355257 33657038 98.0
H3K4me3_no_A3 33700796 32940984 97.7
H3K27Ac_no_A4 39544502 38691396 97.8
d4_H3K27me3_no 25246308 23838650 94.4
H3K4me1_plus_A7 34205143 33534917 98.0
H3K4me3_plus_A8 32855864 32117120 97.8
H3K27Ac_plus_A9 37084614 36349061 98.0

d4_H3K27me3_plus 28265169 26872176 95.1
d9_plus_H3K4me1 20793584 20179985 97.0
d9_plus_H3K4me3 19815874 19224009 97.0
d9_plus_H3K27Ac 21780821 21166524 97.2
d9_plus_H327me3 21435284 20515539 95.7
dTAD_no_H3K4me1 18054874 17443521 96.6
dTAD_plus_H3K4me1 20453274 19808178 96.8
dTAD-Ldb1_no_H3K4me1 18552815 17915469 96.6
dTAD-Ldb1_plus_H3K4me1 18112460 17472574 96.5
HiChIP_d4_plus 89656483
HiChIP_myog_prog_A 94322652
HiChIP_myog_prog_B 91232717
01_d4_Pax3_plusdox 102930219
02_d9_Pax3_shSCR_A 146810118
03_d9_Pax3_shSCR_B 104714913
04_d9_Pax3_shLdb1_A 102275432
05_d9_Pax3_shLdb1_B 144392233
06_d4_Pax3_nodox 134556416
dTAD_Ldb1_nodox 63595450
dTAD_Ldb1_plusdox_A 57611880
dTAD_Ldb1_plusdox_B 60728636

Antibodies

CTCF 07-729 EMD Millipore
H3 (pan) ab1791 Abcam
H3K4me1 ab8895 Abcam
H3K4me3 ab8580 Abcam
H3K27Ac ab4729 Abcam
H3K27me3 07-449 EMD Millipore
LDB1 ab96799 Abcam
LDB1 sc-11198 Santa Cruz Biotechnology
LDB1 sc-365074 Santa Cruz Biotechnology
PAX3 sc-34926 Santa Cruz Biotechnology
SMC1 A300-055A Bethyl

Peak calling parameters

Peak calling was performed using MACS with the following parameters: --bw 300 -p 1e-3. Similar results were obtained by performing peak calling using QESEQ with the following parameters: transcription factors/cofactors -s 100 -c 15 -p 0.01; histones marks -s 100 -c 20 -p 0.001 (replicate 1) -s 100 -c 15 -p 0.001 (replicate 2). To identify the list of high confidence PAX3 and LDB1 peaks we performed 3 independent ChIP-seq experiments and, using the MACS output and the bedtools intersect function, only common regions between 2 experiments were further considered. SMC1 and CTCF peaks were defined as the common peaks among 2 independent ChIP-seq datasets (using bedtools intersect). In addition, peak lists were filtered (intersect –v option) for sites overlapping to peaks detected in the uninduced control ChIP-seq and in the mouse ChIP-seq black-list.

Data quality

For all libraries, the average quality scores for the pass filter reads was ≥Q30. FASTQC was used to assess quality of all libraries and no libraries were discarded due to low quality. All duplicated reads were removed before downstream analyses. Peaks were called using 2 different peak calling algorithms (MACS and QESEQ), which provided similar results. Only high confidence peaks, identified as called in 2 biological replicates, were used for the analyses. ChIP-qPCR was used to validate selected loci. Replicate results for histone marks are provided as Supplementary figure_replicates. HiChIP long-range interactions were called using FDR 1% (q 0.01).

Software

Genomic data analysis: bowtie/2.3.4.1; bedtools/2.27.1; samtools/1.9 ;macs/1.4.1; qeseq/0.2.2; kent/2.4.7; PAVIS2; seqminer/1.3.3; GREAT (Bejerano lab); HiC-pro/2.7.7; FitHiChIP; HiTC; R/3.5.2; Rstudio/1.1.456; pheatmap/1.0.12

Flow Cytometry

Plots

Confirm that:

☒ The axis labels state the marker and fluorochrome used (e.g. CD4-FITC).

☐ The axis scales are clearly visible. Include numbers along axes only for bottom left plot of group (a 'group' is an analysis of identical markers).

☒ All plots are contour plots with outliers or pseudocolor plots.

☒ A numerical value for number of cells or percentage (with statistics) is provided.

Methodology

Sample preparation

Single cells were collected using trypsin, washed with PBS and filtered using a 70micron mesh to remove cell-clumps. If required, cells were fixed with 1% formaldehyde or paraformaldehyde before staining. Cells were incubated with anti-Fc receptor and FBS before adding specific antibodies. After staining, cells were washed with PBS and then resuspended in 5%FBS/PBS supplemented with Propidium Iodide (PI). PI was not used for fixed cells.

Instrument	BD FACS AriaII
Software	FACS Diva
Cell population abundance	>95% determined by post-sort analysis
Gating strategy	1st gate: FSC/SSC; 2nd gate: FSC-w/FSC-h; 3rd gate: SSC-w/SSC-h; 4th gate: PI/PE (select PI negative cells if cells were not previously fixed); 5th gate: PDGFRa-PE/FLK1-APC or FSC/Alexa555. Negative gate were determined in an assay-dependent way (either using non-stained cells, non-induced cells or isotype controls antibodies).

☐ Tick this box to confirm that a figure exemplifying the gating strategy is provided in the Supplementary Information.
